# Mycoremediation of Endosulfan and Its Metabolites in Aqueous Medium and Soil by *Botryosphaeria laricina* JAS6 and *Aspergillus tamarii* JAS9

**DOI:** 10.1371/journal.pone.0077170

**Published:** 2013-10-10

**Authors:** Sivagnanam Silambarasan, Jayanthi Abraham

**Affiliations:** Microbial Biotechnology Laboratory, School of Biosciences and Technology, VIT University, Vellore, Tamil Nadu, India; University of Kansas, United States of America

## Abstract

Microbial degradation offers an efficient and ecofriendly approach to remove toxicants from the contaminated environments. *Botryosphaeria laricina* JAS6 and *Aspergillus tamarii* JAS9 were capable of degrading endosulfan and their metabolites which were isolated through enrichment technique. Both the strains were able to withstand an exposure of 1300 mg/L and showed luxuriant growth at 1000 mg/L of endosulfan. The change in pH in the culture broth was from 6.8 to 3.4 and 3.8 during growth kinetic studies of JAS6 and JAS9 strains, respectively upon biological degradation of endosulfan. The degradation of endosulfan by JAS6 and JAS9 strains were examined by HPLC. The biodegradation rate constant (k) and the initial concentration were reduced by 50% (DT_50_) which was determined by first and pseudo first order kinetic models. In the present investigation it has been revealed that *Botryosphaeria laricina* JAS6 and *Aspergillus tamarii* JAS9 possessing endosulfan degrading capability are being reported for the first time. These findings confirm the degradation of endosulfan by JAS6 and JAS9 strains which were accompanied by significant reduction in the toxicity and could be used as remedial measure in contaminated environments.

## Introduction

Endosulfan(6,7,8,9,10,10-hexachloro-1,5,5a,6,9,9a-hexahydro-6,9-methano-2,4,3-benzodioxathiepin-3-oxide) is a chlorinated cyclodiene pesticide (insecticide and acaricide) classified under the category of persistent organic pollutant. Its commercial preparation consists of a mixture of two isomers approximately 70% α-endosulfan and 30% β-endosulfan. Endosulfan has been extensively used for over 30 years on a variety of vegetables, cereals, fruit and cotton [1]. Endosulfan application continues in some countries but has been banned in the European Union since 2006 [2] and from 2011 in India. Endosulfan and its metabolites are persistent in the environment with an estimated half life of 0.7-6 years [3]. The impact of endosulfan and its toxic metabolites in the environment result in contamination and persistence leading to bioaccumulation and biomagnification.

Endosulfan is extremely toxic to fish and aquatic invertebrates and the major symptoms of its poisoning is the stimulation of the central nervous system, but prolonged exposure to endosulfan affects the blood chemistry, kidney, liver, reduction in sperm count, and mutagenic effects [4-7]. These deleterious effects on health and environment concerns have led to an interest in detoxiﬁcation of endosulfan and its metabolite in the environment.

The bioremediation of endosulfan is receiving serious attention as an alternative to other methods such as incineration and landfill [8]. In biodegradation study, the microorganisms are able to break down the recalcitrant organic compounds to obtain chemical energy; hence organic compound can serve as carbon, energy and nutrient sources for microbial growth [8]. The major metabolites of endosulfan formed during microbial degradation are endosulfan diol, endosulfan sulfate, endosulfan ether, endosulfan hydroxyether, endosulfan lactone and endosulfan dialdehyde [9-11]. Among which the production of endosulfan sulfate is the major toxic metabolite that persists longer in soils and has bioaccumulation potential.

There have been many research works in determining the microbial degradation of endosulfan in aqueous medium and soil which includes *Achromobacter xylosoxidans* strain C8B [12], *Bordetella* sp. B9 [13], *Klebsiella oxytoca* [10], *Bacillus* sp. [14], *Pandoraea* sp. [3], *Micrococcus* sp. [15], *Aspergillus niger* [16,17], *Aspergillus terreus* and *Cladosporium oxysporum* [18], *Fusarium ventricosum* [3], *Mucor thermohyalospora* [19], *Phanerochaete chrysosporium* [20], *Trichoderma harzianum* [21]. In the present study, two fungal strains were isolated using enrichment technique which was able to degrade α-endosulfan, β-endosulfan and major toxic metabolite endosulfan sulfate. To the best of our knowledge, this is the first report on degradation of endosulfan by *Botryosphaeria laricina* JAS6 and *Aspergillus tamarii* JAS9.

## Materials and Methods

### Chemicals

Technical grade endosulfan (35% emulsified preparation) was used in this study which was obtained from Hindustan Insecticides Ltd., Kerala, India. Analytical grade α-endosulfan (99% purity), β-endosulfan (99% purity) and endosulfan sulfate (99% purity) were used as standards procured from Sigma Aldrich (St. Louis, MO, USA). Chromatographic grade acetonitrile and ethyl acetate were purchased from SD Fine Chem Limited (India). All other reagents used in this study were of analytical reagents quality. 

### Enrichment and isolation of fungal strain

The soil sample was collected from *Abelmoschus esculentus* (Ladies finger) grown agricultural field of Vellore in Tamil Nadu, India with prior permission from owner of the field. The fields have been exposed to endosulfan for a period of 5 years for the cultivation of *Abelmoschus esculentus*. The soil was air dried at room temperature in the laboratory and passed through a 2 mm sieve. The chemical properties of the soil were studied at Shri AMM Murugappa Chettiar Research Centre, Taramani, Chennai, India (Table 1).

**Table 1:** Chemical properties of the soil used in the experiment.

Properties	Soil sample
**pH**	7.87
**EC**	0.3
**Organic Carbon**	1.23 kg/hectare
**Organic Carbon**	0.55 %
**Nitrogen**	316.76 kg/ hectare
**Phosphorus**	18.70 kg/ hectare
**Potassium**	226.39 kg/ hectare
**Calcium**	395.47 mg/kg
**Magnesium**	163.12 mg/kg
**Sodium**	102.04 mg/kg
**Iron**	6.36 mg/kg
**Manganese**	7.43 mg/kg
**Copper**	1.54 mg/kg
**Zinc**	0.9 mg/kg
**Sulfate**	14.33 mg/kg
**Humus**	217.87 kg/ hectare
**Total Minerals**	561.86 kg/ hectare

The enrichment culture technique was employed for the isolation of efficient fungal strain capable of degrading endosulfan. 5 g of soil sample was inoculated in 100 ml of Czapek Dox broth containing (in g/L) yeast extract 3; peptone 10; dextrose 2; pH 6.8 and endosulfan 35 mg/L. The culture was incubated at 28 ± 2 °C at 120 rpm for 5 d. The enrichment culture was serially diluted and spread on modified Czapek Dox medium containing (in g/L) sucrose 30; magnesium sulfate 0.5; sodium nitrate 2; dipotassium sulfate 0.35; ferrous sulfate 0.001; agar agar 30; pH 6.8 and 35 mg/L of endosulfan. After 5 d of incubation at 30 °C, colonies were purified and selected for further study. 

### Gradient plate

The endosulfan gradient plate was prepared by adding a base layer of 20 ml Czapek Dox agar to a petri plate tilted at an angle of 30° and it was allowed to solidify. Onto the solidified base, 20 ml of agar containing endosulfan (1000 mg/L) was poured to give an endosulfan gradient across the plate surface. The spore suspensions was prepared in 0.1% triton X-100 from the isolated fungal strain and streaked on the endosulfan gradient plate using a sterile cotton swab. The plates were incubated at 30 °C for 8 d and length of growth was recorded [16].

### Identification of highly efficient fungal strains

The fungal strains exhibiting excellent growth on gradient plate was identified by 18S rRNA sequence analysis [22]. The fungal genomic DNA was isolated by using AMpurE Fungal gDNA Mini kit. In this kit detergent and other non corrosive chemicals are used to break open the cellulosic cell wall and plasma membrane to extract DNA from fungal cells. The 18S r RNA gene was amplified by polymerase chain reaction (PCR) using the universal primers ITS1 and ITS4. PCR reaction mix of 50 µl final volume contained: 50 ng sample gDNA, 100 ng forward primer, 100 ng reverse primer, 2 µl dNTP’s mixture (10 mM), 5 µl 10X Taq polymerase buffer, 3 U Taq polymerase enzyme and PCR grade water to make up the volume. Amplified PCR product was sequenced by using ABI3730xl genetic analyzer (Amnion Biosciences Pvt. Ltd. Bangalore, India). The sequencing result was submitted to the GenBank National Center for Biotechnology Information (NCBI) database. 

### Minimum inhibitory concentration and growth kinetics

The fungal strain showing the greatest length along the gradient was selected in order to check the minimum inhibitory concentration (MIC) of endosulfan. M1 medium was used for the MIC assay and it is composed (g/L) of sodium nitrate 2; potassium chloride 0.5; magnesium sulfate 0.5; glucose 10; ferric chloride 10 mg; barium chloride 0.2; calcium chloride 0.05 and pH 6.8. MIC was carried out in 100 ml M1 medium amended with increasing concentrations of endosulfan from 100 to 1500 mg/L. The flasks were inoculated with 1 ml of fungal spore suspension (10^8^ spores/ml) prepared in 0.1% Triton X-100 [16] and incubated on a rotary shaker at 120 rpm at 30 ± 2 °C for 10 d. After incubation mycelial growth was observed in the flasks and the MIC was noted as the concentration of endosulfan resulting in complete inhibition of mycelial growth in flasks. Mycelial mass was separated from each flask by filtration using Whatmann filter paper and washed with deionized water. The dry weight of fungal biomass was determined by drying at 80 °C in pre weighed aluminum foil cups. 

 The growth kinetics was studied in modified Czapek Dox broth with and without endosulfan (1000 mg/L). The study was performed in triplicates. One milliliter of spore suspension (10^8^ spores/ml) was inoculated into a series of flasks containing Czapek Dox broth. The flasks were incubated at 30 ± 2 °C on a rotary shaker at 120 rpm. One flask from each series was removed at 12, 24, 48, 72, 96 and 120 h intervals. After incubation mycelial mass from each flask was filtered and dry weight was estimated. Organic acid production in the Czapek Dox broth with and without endosulfan was determined by centrifuging the cultures at 10,000 rpm for 10 min. The supernatants were filtered through a 0.22 µm filter membrane and the filtrate was injected to High Performance Liquid Chromatography (HPLC). The mobile phase consisting of 0.1% phosphoric acid with a constant flow rate of 1 ml/min. Elutes were analyzed at 210 nm and identified by comparing the retention time with that of the pure organic acids. 

### Biodegradation of endosulfan and its major metabolites in aqueous medium

The biodegradation experiments in aqueous medium were conducted in 250 ml Erlenmeyer flask that contained 100 ml of M1 medium and supplemented with 1000 mg/L of endosulfan as carbon source. One milliliter of fungal spore suspension (10^8^ spores/ml) was inoculated into a series of flasks and uninoculated flask was maintained as control. The flasks were then incubated at 30 ± 2 °C on a rotary shaker at 120 rpm. The samples were taken at 1 d interval and the concentrations of endosulfan and its major metabolites were determined by using HPLC.

### Biodegradation of endosulfan and its major metabolites in soil microcosm

In order to determine the ability of fungal strain to degrade endosulfan and its metabolites the soil sample from which fungus was isolated was utilized as culture medium for degradation studies. Two soil microcosm treatments were carried out with (1) addition of pesticide, fungal spore and nutrients (carbon, nitrogen and phosphorus) and (2) addition of pesticide and fungal spore without nutrients (control). Before using the soil for degradation studies, it was sterilized by autoclaving three times each at 4 h interval for 30 min at 121 °C. 30 ml of solution containing fungal spore, nitrogen, phosphorus, glucose and 1000 mg/L of endosulfan were added to 250 ml Erlenmeyer flask which contained 100 g of sterilized soil. The sources of carbon, nitrogen and phosphorus were glucose, ammonium sulphate and dipotassium hydrogen phosphate, respectively. The amounts of carbon, nitrogen and phosphorus were calculated using the relationship C/N/P (100:10:1) [23,24]. All the flasks were incubated at 30 °C for 10 d and soil samples were taken at 1 d interval regularly for the determination of endosulfan and its major metabolites concentration. 

### Extraction of endosulfan and its metabolites from aqueous medium and soil microcosm

Endosulfan and its metabolites were extracted from aqueous medium with addition of equal volume of acetonitrile and from soil microcosm by adding 50 ml of acetonitrile to 10 g of soil sample, the mixture was shaken for 1 h at 200 rpm on a rotary shaker and then centrifuged. The supernatant was decanted into a glass beaker and the organic solvent was concentrated in a water bath at 35 °C. The extracted samples were used for the detection of endosulfan and its metabolites by HPLC [25].

### Analytical procedure

The extracted samples were analyzed by HPLC (Waters 1525 binary HPLC pump, Milford, USA) on a Symmetry C_18_ column (Waters 5 µm, 4.6 mm × 150 mm). The operating conditions were as follows: mobile phase comprised of acetonitrile:water (65:35), ﬂow rate 1 ml/min, duration of cycle 20 min, detector wavelength 214 nm and injection volume 25 µl [12]. 

Infrared (IR) spectra of the parent compound (endosulfan) and sample after degradation of endosulfan with fungal strains were recorded in the frequency range of 4,000-400 cm^-1^ with a Fourier transform infrared (FTIR) spectrophotometer (8400 Shimadzu, Japan, with Hyper IR-1.7 software for Windows) with a helium-neon laser lamp as a source of IR radiation. Pressed pellets were prepared by grinding the extracted samples with potassium bromide in a mortar with 1:100 ratio and analyzed in the region of 4,000-400 cm^-1^ at a resolution of 4 cm^-1^. 

### Kinetic studies

The degradation rate constant (k) was determined using the algorithm ln *C_t_/C*_*o*_ = *e* ^-kt^ (first order kinetic model) and ln *C*_*t*_ *=* - *kt* + ln *C*_*o*_ (pseudo first order), where *C*_*o*_ is the amount of pesticide in M1 medium or soil at time zero, *C*_*t*_ is the amount of pesticide in M1 medium or soil at time *t*, and *k* and *t* are the rate constant (d^-1^) and degradation period in days, respectively. The theoretical DT_50_ values were calculated from the linear equation obtained from the regression between ln (*C_t_/C*_*o*_) for first order model and ln *C*_*t*_ for pseudo first order model of the chemical data and time. 

## Results and Discussion

### Isolation and characterization of endosulfan degrading fungal strains

**Figure 1:**
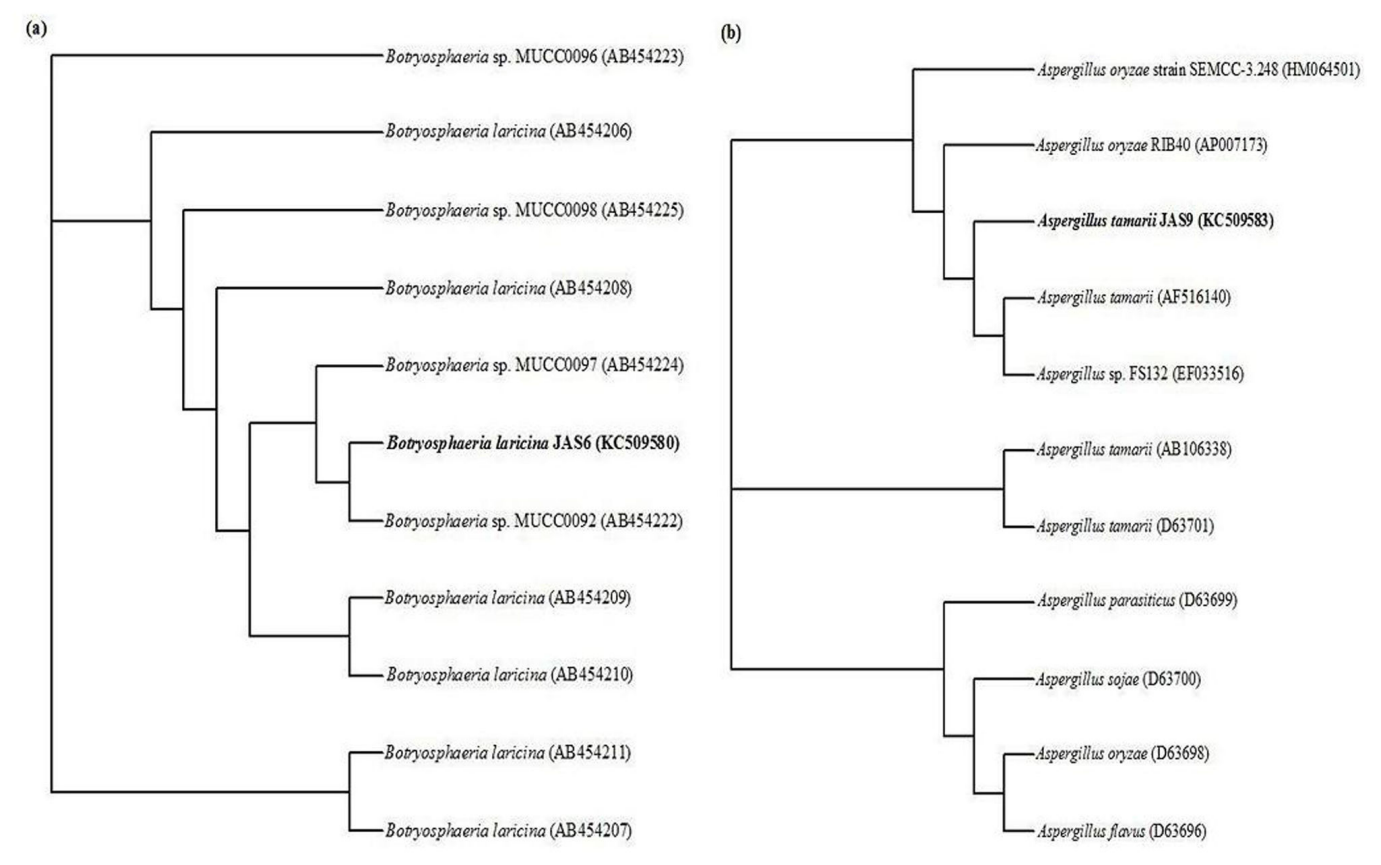
Phylogenetic analysis of 18S rRNA gene sequences (a) between *Botryosphaeria laricina* JAS6 and reference sequences; (b) *Aspergillus tamarii* JAS9 and reference sequences retrieved from NCBI GenBank constructed through neighbor joining method.

Two morphologically different fungal strains JAS6 and JAS9 were isolated through enrichment culture technique from endosulfan spiked soil sample which was collected from *Abelmoschus esculentus* fields and further the isolated strains were purified by repeated streaking on modified Czapek Dox agar medium. The gradient plate assay was used to screen the potential strain for endosulfan degradation as reported by Bhalerao and Puranik [16]. The gradient plate method is a simple and reliable technique which provides a wide range of pesticide concentration across the gradient. Growth of fungal strains on the gradient plate was obtained as the length in centimeters across the endosulfan gradient. The two strains had the greatest length of growth about > 5.6 cm along the gradient and the highest tolerance was selected for the further studies. The JAS6 and JAS9 strains were identified based on 18S rRNA gene sequences analysis. The nucleotides obtained were compared with reference sequences by using BLAST similarity searches and the closely related sequences were obtained from GenBank. The phylogenetic analysis (Figure 1) indicated that the strains JAS6 and JAS9 cluster with *Botryosphaeria laricina* and *Aspergillus tamarii*, respectively exhibiting 99% similarity. The GenBank accession number for the 18S rRNA sequence of *Botryosphaeria laricina* JAS6 and *Aspergillus tamarii* JAS9 isolates were KC509580 and KC509583, respectively. Earlier works have reported that *Aspergillus tamarii* as an endophyte of *Coscinium fenestratum* [26] and certain compound like aﬂatoxin [27], fumigaclavine A [28], cyclopiazonic acid [29], kojic and itaconic acids [30] have been produced from *Aspergillus tamarii*. However, this is the first report on *A.tamarii* in bioremediation studies. *Botryosphaeria* sp., a ligninolytic ascomyceteous fungus, was reported capable of growing on high concentrations of the lignin like aromatic compound, 3,4-dimethoxybenzyl alcohol and appeared to have potential for bioremediation of aromatic compounds [31,32]. 

### MIC and growth kinetics of JAS6 and JAS9 strains

The results obtained from gradient plate assay were confirmed the growth of JAS6 and JAS9 strains in M1 medium with varying concentrations of endosulfan for MIC determination. Both the strains could tolerate up to 1300 mg/L and showed luxuriant growth at 1000 mg/L of endosulfan. Bhalerao and Puranik [16] reported *Cunninghamella echinulata*, *Aspergillus oryzae*, *Aspergillus niger* and *Emericella nivea* tolerated 300, 275, 400 and 325 mg/L of endosulfan, respectively. However, in this present investigation potential strains of JAS6 and JAS9 were able to tolerate higher concentration of endosulfan dosage than observed in earlier studies. 

**Figure 2:**
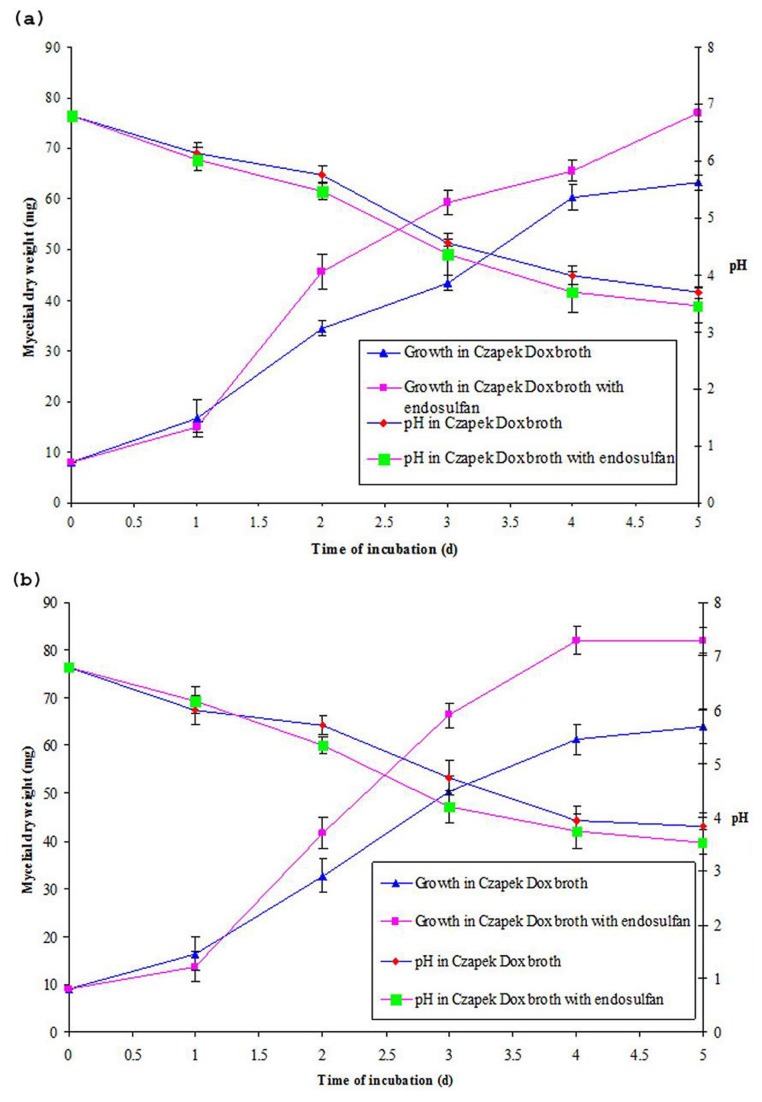
Fungal growth monitored in terms of biomass and culture pH in media. (a) *Botryosphaeria laricina* JAS6 gowth in presence and absence of endosulfan; (b) *Aspergillus tamarii* JAS9 growth in presence and absence of endosulfan. The interpolated curves are arbitrary and are there to guide the eye only. Each value is the mean of three replicates, with error bars representing the standard deviation of the mean.

The growth kinetics of *Botryosphaeria laricina* JAS6 and *Aspergillus tamarii* JAS9 were studied in the presence and absence of endosulfan which was indicated by the increase in mycelial mass with respective time intervals are presented in the Figure 2. At the initial stages, both strains showed poor growth in the presence of endosulfan but after acclimatization to endosulfan, the strains grew rapidly and exhibited luxurious growth. After incubation, the amount of biomass produced in the culture medium with endosulfan was much higher when compared to the growth without endosulfan and this could be attributed to the fact that the availability of additional source of carbon or sulfur upon degradation of endosulfan in the medium [16]. However, in our study the increasing mycelial biomass was observed at 2 d of incubation. In addition as a consequence of *Botryosphaeria laricina* JAS6 and *Aspergillus tamarii* JAS9 growth, pH in the culture medium rapidly decreased which may be correlated, to the change in pH of the culture medium which decreased drastically to acidic range with the increase in metabolic activities during mineralization of endosulfan. This reduction in pH in medium with fungal strains might be due to dehalogenation of endosulfan and subsequent formation of acidic substances. Further, the production of organic acids in the medium was confirmed by HPLC analysis. JAS6 strain produced 16,531 µg/ml of oxalic acid in the culture medium without endosulfan and 14,378 µg/ml of oxalic acid with endosulfan spiked in culture medium, whereas JAS9 strain produced 17,871 µg/ml of oxalic acid in the culture medium without endosulfan and 15,643 µg/ml with endosulfan. These results were consistent with previous findings that concluded that the decrease in pH might be due to the formation of HCl or organic acids by microorganisms [3,14,33,34]. 

### Biodegradation of endosulfan in aqueous medium and soil

Biodegradation of endosulfan in aqueous medium and soil by the efficient fungal strains *Botryosphaeria laricina* JAS6 and *Aspergillus tamarii* JAS9 was investigated using HPLC and FTIR. Recovery experiments were conducted in the M1 medium and soil, inorder to analyze the extraction efficiency of the methods employed. Different known concentrations of endosulfan was spiked in 50 ml of the M1 medium (100, 200, and 300 mg/L) and 50 g of soil (100, 200, and 300 mg/L). Average recoveries of endosulfan from the M1 medium at levels of 100, 200, and 300 mg/L were measured to be 96.3±4.6, 95.6±4.3, and 97.8±1.3 %, respectively. The corresponding recoveries from the soil at levels of 100, 200, and 300 mg/L were 94.6±3.3, 93.8±3.5, and 92.2±0.9 %, respectively. These data indicated that HPLC for endosulfan determination has a high accuracy, and the extraction procedures were efficient in extracting the endosulfan residues from the M1 medium and soil. By using authentic standards (analytical grade), HPLC analysis demonstrated the degradation of endosulfan in the aqueous medium by *Botryosphaeria laricina* JAS6 with the formation of β-endosulfan and endosulfan sulfate metabolites, whereas *Aspergillus tamarii* JAS9 degraded the endosulfan and yielded three metabolites such as α-endosulfan, β-endosulfan and endosulfan sulfate. The degradation experiment was conducted in soil with and without addition of nutrients for 10 d. Endosulfan was not mineralized on the 10 d of incubation in the soil inoculated with JAS6 and JAS9 strains devoid of nutrients. In contrast JAS6 and JAS9 strains degraded endosulfan from soil ammended with nutrients and metabolites such as α-endosulfan and β-endosulfan were detected. The three different endosulfan metabolites (α-endosulfan, β-endosulfan and endosulfan sulfate) were found to accumulate in the aqueous medium and soil microcosm studies. Previous researchers have reported endosulfan as a sole carbon source [10,14,35] or as a sulfur source for microbial growth [10,36]. In this sudy JAS6 and JAS9 strains could utilize endosulfan as the sole carbon and energy source. These efficient fungal strains were able to tolerate 1000 mg/L of and in degradation experiments JAS6 and JAS9 strains degraded endosulfan and its metabolite α-endosulfan, β-endosulfan in soil with nutrients at 10 d of incubation. 

The biodegradation of endosulfan and its metabolites rate constant (k) and the initial concentration was reduced by 50% (DT_50_) were determined by first and pseudo first order kinetic models (Table 2). Kinetics data showed that the β-endosulfan and endosulfan sulfate accumulated in aqueous medium was degraded by JAS6 strain which was characterized by the rate constant (k) of 0.164 d^-1^ (β-endosulfan) and 0.924 d^-1^ (endosulfan sulfate). The times within which the initial pesticide concentration was reduced by 50% (DT_50_) was 4.2 d (β-endosulfan) and 0.7 d (endosulfan sulfate). The two endosulfan metabolites, α, β-endosulfan appeared in the soil microcosm study was degraded by JAS6 with a rate constant of 0.183 d^-1^ and 0.414 d^-1^, respectively; and DT_50_ was 3.7 d and 1.6 d, respectively. The α-endosulfan, β-endosulfan and endosulfan sulfate metabolites were detected in the aqueous medium was degraded by JAS9 strains, which was characterized with a rate constant of 0.395 d^-1^, 0.312 d^-1^, 0.242 d^-1^ and DT_50_ was 1.754 d, 2.221 d, 2.864 d, respectively. JAS9 strain degraded endosulfan in soil when amended with nutrients, two metabolites such as α-endosulfan and β-endosulfan were produced which was further degraded by the same strain with a rate constant of 0.302 d^-1^ (α-endosulfan), 0.200 d^-1^ (β-endosulfan) and DT_50_ was 2.2 d (α-endosulfan) and 3.4 d (β-endosulfan). 

**Table 2:** Kinetic parameters for the degradation of endosulfan and its metabolites by *Botryosphaeria laricina* JAS6 and *Aspergillus tamarii* JAS9.

Treatments	First order kinetic			Pseudo first order kinetic		
	***k* (d^-1^)**	**DT_50_**	***R*^2^**	***k* (d^-1^)**	**DT_50_**	***R*^2^**
**M1 medium + beta endosulfan + JAS6**	0.164	4.226	0.867	0.164	4.226	0.868
**M1 medium + endosulfan sulfate + JAS6**	0.924	0.750	0.907	0.924	0.750	0.907
Soil + N + alpha endosulfan + JAS6	0.183	3.787	0.827	0.183	3.787	0.826
Soil + N + beta endosulfan + JAS6	0.414	1.674	0.979	0.414	1.674	0.979
**M1 medium + alpha endosulfan + JAS9**	0.395	1.754	0.956	0.395	1.754	0.956
**M1 medium + beta endosulfan + JAS9**	0.312	2.221	0.833	0.313	2.214	0.833
**M1 medium + endosulfan sulfate + JAS9**	0.242	2.864	0.845	0.242	2.864	0.847
Soil + N + alpha endosulfan + JAS9	0.302	2.295	0.899	0.301	2.302	0.899
**Soil + N + beta endosulfan + JAS9**	0.200	3.465	0.975	0.200	3.465	0.975

Note: N, nutrients such as glucose, (NH_4_)_2_SO_4_ and K_2_HPO_4_.

**Figure 3:**
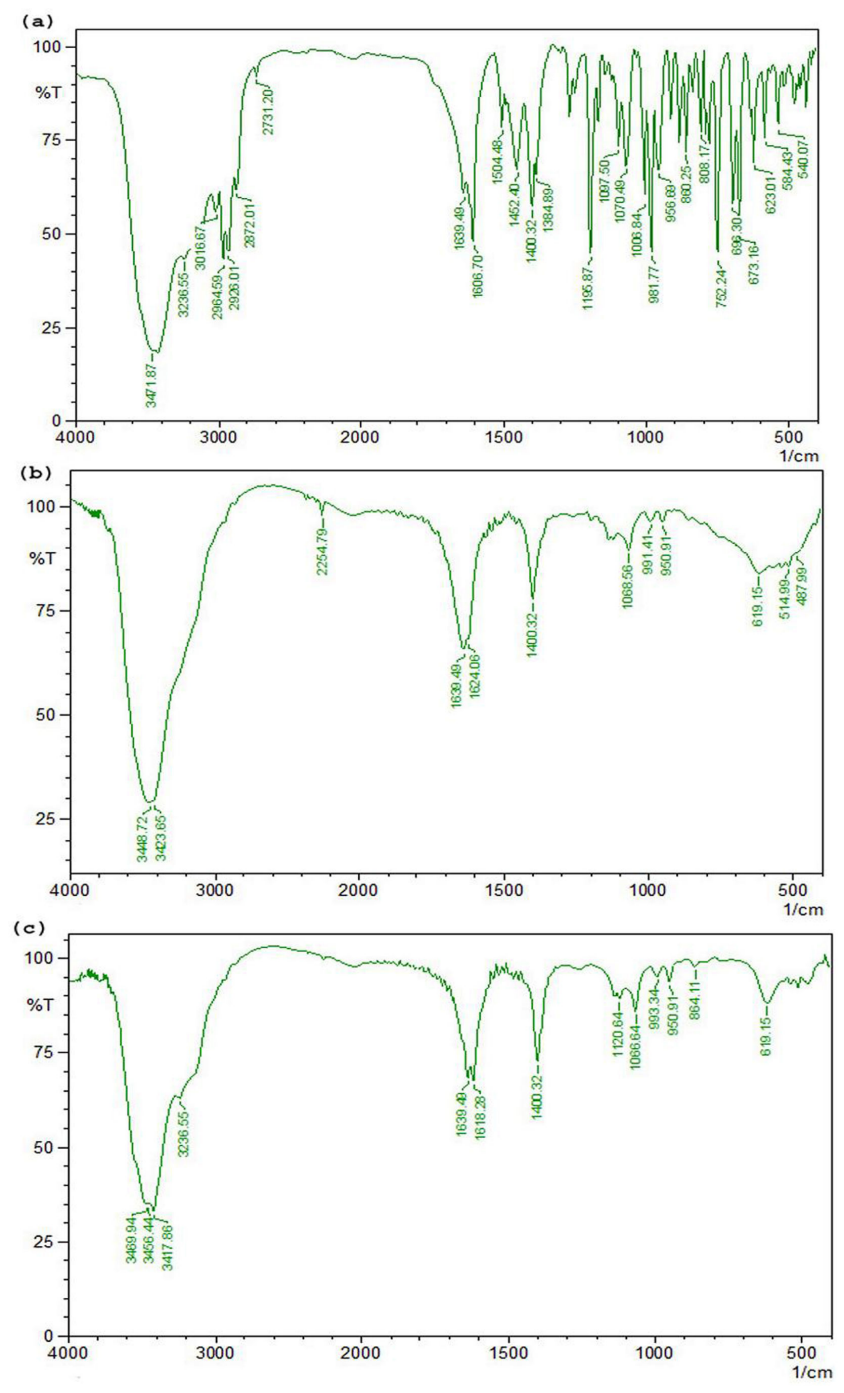
FTIR spectrum of endosulfan at (a) standard condition; (b) FTIR spectrum for the degradation of endosulfan and its metabolites in aqueous medium by JAS6; (c) FTIR spectrum for the degradation of endosulfan and its metabolites in aqueous medium by JAS9.

The FTIR analysis revealed the various structural changes of endosulfan (Figure 3). Comparison of FTIR spectrum of control with extracted metabolites after complete degradation clearly indicated the biodegradation of endosulfan and its metabolites. The infrared spectrum of endosulfan degraded sample in the aqueous medium by JAS6 and JAS9 showed bands at 1400 cm^-1^ are the characteristics COOH group. The acid dimer band was seen from endosulfan degraded samples in the aqueous medium by JAS6 and JAS9 at 950 cm^-1^. The formation of acid group in the final degraded samples confirms the degradation of endosulfan. This result was well in accordance with the observation of Guerin [37] who reported that major metabolite of endosulfan sulfate was converted to non-recoverable acidic forms.

In a study conducted by Bhalerao and Puranik [16], regarding endosulfan degradation by *Aspergillus niger* they observed endosulfan sulfate which was the final metabolite that persisted up to 90 h in aqueous medium. Sutherland et al. [33] had demonstrated the formation and persistence of endosulfan sulfate during the bioremediation of endosulfan. In the present study, the formation of endosulfan sulfate as a major metabolite was eminent and it was subsequently utilized during endosulfan degradation. This demonstrates the active role of JAS6 and JAS9 in the degradation of endosulfan. Li et al. [25] demonstrated that *Achromobacter xylosoxidans* CS5 could degrade more than 24.8 mg/L of α-endosulfan and 10.5 mg/L β-endosulfan after 8 days of incubation in aqueous medium, with the formation of major metabolites as endosulfan diol and endosulfan ether. The three bacterial strains, *Pseudomonas aeruginosa, P. spinosa*, and *Burkholderia cepacia*, degraded both α-endosulfan and β-endosulfan more than 90% of the spiked amount (100 mg/L) in the broth within 14 d of incubation [38]. However, in our study both the fungal strains, *Botryosphaeria laricina* JAS6 and *Aspergillus tamarii* JAS9 efficiently degraded endosulfan and its major metabolites α-endosulfan and β-endosulfan in soil within 10 d of incubation. The fungal strains, *Chaetosartorya stromatoides*, *Aspergillus terricola*, and *Aspergillus terreus* degraded both α-endosulfan and β-endosulfan up to 75% of 100 mg/L in the broth within 12 d [39]. Singh and Singh [12] showed that *Achromobacter xylosoxidans* strain C8B could degrade 94.12% of α-endosulfan, 84.52% of β-endosulfan and also it degrade 80.10% of endosulfan sulfate. 

## Conclusions

In this report, endosulfan and its metabolites degrading *Botryosphaeria laricina* JAS6 and *Aspergillus tamarii* JAS9 strains were isolated from endosulfan spiked soil. The two strains JAS6 and JAS9 were able to tolerate elevated levels endosulfan as high as 1300 mg/L and grew well up to 1000 mg/L. The degradation of endosulfan by these strains were simple, rapid and highly effective, which was confirmed with appearance of endosulfan metabolites. Another important feature which is worth mentioning is that these particular strains were capable of degrading endosulfan sulfate. Degradation of this compound by the same strain that degrades endosulfan is very important because endosulfan sulfate is a major toxic metabolite that persists longer in soil. Kinetic studies reveal a very good compliance with the ﬁrst and pseudo ﬁrst order model. *Botryosphaeria laricina* JAS6 and *Aspergillus tamarii* JAS9 could be employed as potential microbes for the safe treatment of endosulfan contaminated water and soil due to its high degradation efficiency.
